# Structural Study of Sulfur-Added Carbon Nanohorns

**DOI:** 10.3390/ma15103412

**Published:** 2022-05-10

**Authors:** Ysmael Verde-Gómez, Elizabeth Montiel-Macías, Ana María Valenzuela-Muñiz, Ivonne Alonso-Lemus, Mario Miki-Yoshida, Karim Zaghib, Nicolas Brodusch, Raynald Gauvin

**Affiliations:** 1 Tecnológico Nacional de México/I.T. de Cancún, Av. Kabah km. 3, Cancún 77500, Q.Roo., Mexico; elizabethmontielmacias@hotmail.com (E.M.-M.); ana.vm@cancun.tecnm.mx (A.M.V.-M.); 2 CONACyT-CINVESTAV Unidad Saltillo, Sustentabilidad de los Recursos Naturales y Energía, Av. Industria Metalúrgica, Parque Industrial Saltillo-Ramos Arizpe, Ramos Arizpe 25900, Coah., Mexico; ivonne.alonso@cinvestav.edu.mx; 3 Centro de Investigación en Materiales Avanzados S.C., Av. Miguel de Cervantes 120, Chihuahua 31136, Chih., Mexico; mario.miki@cimav.edu.mx; 4 Department of Chemical and Materials Engineering, Concordia University, 1515 Rue Sainte-Catherine O, Montréal, QC H3G 2W1, Canada; karim.zaghib@concordia.ca; 5 Department of Mining and Materials Engineering, McGill University, 3610 University Street, Montréal, QC H3A 0C5, Canada; nicolas.brodusch@mcgill.ca (N.B.); raynald.gauvin@mcgill.ca (R.G.)

**Keywords:** carbon nanohorns, sulfurated nanostructures, iron sulfide nanoparticles, chemical vapor deposition

## Abstract

In the past few decades, nanostructured carbons (NCs) have been investigated for their interesting properties, which are attractive for a wide range of applications in electronic devices, energy systems, sensors, and support materials. One approach to improving the properties of NCs is to dope them with various heteroatoms. This work describes the synthesis and study of sulfur-added carbon nanohorns (S-CNH). Synthesis of S-CNH was carried out by modified chemical vapor deposition (m-CVD) using toluene and thiophene as carbon and sulfur sources, respectively. Some parameters such as the temperature of synthesis and carrier gas flow rates were modified to determine their effect on the properties of S-CNH. High-resolution scanning and transmission electron microscopy analysis showed the presence of hollow horn-type carbon nanostructures with lengths between 1 to 3 µm and, diameters that are in the range of 50 to 200 nm. Two types of carbon layers were observed, with rough outer layers and smooth inner layers. The surface textural properties are attributed to the defects induced by the sulfur intercalated into the lattice or bonded with the carbon. The XRD patterns and X-ray microanalysis studies show that iron serves as the seed for carbon nanohorn growth and iron sulfide is formed during synthesis.

## 1. Introduction

Nanostructured carbon (NC) materials have the potential to be used in many fields due to their unique and tunable properties. Synthesis methods and applications of some NCs such as carbon nanotubes, fullerenes, and recently graphene have been widely reported. However, horn-type carbon nanomaterials are less studied and therefore less reported. Initially, carbon nanohorns (CNH) were observed as aggregates of other NCs, and in 1999, Iijima et al. reported their synthesis [1]. CNHs differ from other carbon allotropes by their cone-shaped tips and sp^2^-hybridized carbon structure [2]. In this regard, the single-walled carbon nanohorn (SWCNH) with dahlia-like shape is one of the most studied of this family [3]. SWCNHs are considered to be a promising material in fields such as nanomedicine [4], energy [5], absorbents, and catalysis [6]. However, the development and study of CNHs has been slow, mainly due to spherical agglomerates which form during their synthesis process, hindering their functionalization and use of their entire surface [7]. Notwithstanding the foregoing, the production of CNHs can be cheap and easily scalable, and sometimes a metal precursor is not required [8]. Some companies have CNH production facilities, such as the NEC Corporation using laser ablation [9], Carbonium SRL using Joule heating method [10], and EEnano Tech Ltd. using arc discharge method [11].

The most outstanding properties of pristine CNHs over other carbon allotropes are their unique geometry, their ability to be produced at room temperature, high thermal and chemical stability, high porosity, and roughness, which makes them more reactive than nanostructures such as carbon nanotubes [12,13,14,15]. On the other hand, theoretical studies show that the electrostatic dipole moment increases with the number of carbon atoms in the conical tip, which also improves the reactivity of these nanostructures [16].

On the other hand, it is well known that NCs doped with heteroatoms (e.g., S, N, B, and P) enhance their properties [17]. In particular, the doped carbons have proven to be versatile functional materials with a wide range of potential applications, including heterogeneous catalysts for oxygen reduction reactions (ORR) [18,19,20], anodes for Li-ion batteries [21,22,23], cathodes for lithium-oxygen batteries [24], supercapacitors [25], adsorbents for hydrogen storage and CO_2_ capture [26], adsorption of heavy metals and toxic gases [27], and desulfurization of diesel and crude oil [28] among others.

However, the synthesis of heteroatom-doped CNHs has not been explored despite the potential they could have in multiple applications. For example, sulfur-doped CNHs have shown to be a promising bifunctional catalyst for water splitting [29], while N-O-doped CNHs have high stability and good production rates for the electrosynthesis of hydrogen peroxide [30,31]. Furthermore, it has been reported that S-doped CNH and N-doped CNH have reliable performance with regard to oxygen evolution and reduction reactions, respectively [32,33]. In addition, N-doped SWCNH is a low-cost and high-performance electrode material for Lithium-sulfur battery applications [34].

Thus, the synthesis of heteroatom-doped CNHs by easy and scalable methods is attractive. This work presents the synthesis of novel sulfur-added multiwalled carbon nanohorns (S-CNH) by a modified one-step chemical vapor deposition method, evaluating the effect of the gas flow rate and the synthesis temperature on the physicochemical properties of the S-CNH.

## 2. Materials and Methods

Synthesis of the sulfur-added carbon nanohorns (S-CNH) was conducted by a Modified Chemical Vapor Deposition method (m-CVD) in a tubular furnace. A Vycor^®^ tube (96% silica glass, Corning, Corning, NY, USA) was used as a substrate for deposition of the S-CNH. A precursor solution of toluene (99.8% Sigma-Aldrich, St. Louis, MO, USA) and thiophene (99% Alfa Aesar, Haverhill, MA, USA) (4:1 %vol.) served as the carbon and sulfur source, respectively. Ferrocene (98% Sigma-Aldrich, St. Louis, MO, USA) was added as an organometallic precursor (19 g/L) that acts as a source of Fe nanoparticles on which the CNH grows. The precursor solution was preheated until vaporization before its introduction into the furnace. The furnace temperature was 800 and 900 °C, while the carrier gas (argon) flow rate was 0.5 and 1 L/min. After the precursor solution was completely vaporized (50 mL), the tube was kept under an inert atmosphere until it cooled to room temperature.

After the synthesis, the samples were treated to remove residual iron and amorphous carbon using a reflux system with concentrated nitric acid for 12 h; the S-CNH-acid solution was continuously stirred. Following this procedure, the S-CNHs were recovered by filtration and washed with distilled water to remove all residual acid. Finally, the samples were dried overnight at 80 °C. The samples were labeled by the following code: SCNA8, SCNA9, SCNB8, and SCNB9, where A is carrier gas flow of 1.0 L/min, and B is carrier gas flow of 0.5 L/min; 8 and 9 correspond to the synthesis temperature of 800 °C and 900 °C, respectively.

The structural properties were analyzed by X-ray diffraction (XRD) and Raman spectroscopy. XRD analysis was performed with a Bruker diffractometer, model D8 Advance using Bragg–Brentano scan mode, LIXEYE detector, a step size of 0.01°, and step time of 5 s in a 2θ range from 10 to 85°. Raman analysis was performed using a Micro Raman Horiba spectrometer, Labram model, with a He-Ne laser (632.8 nm). The morphology and nanostructure of the samples were characterized by transmission electron microscopy (TEM) with a JEOL JEM 2200FS + CS at an accelerating voltage (E0) of 200 kV. A HITACHI SU-8230 (Hitachi, Tokyo, Japan) was used for scanning electron microscopy (SEM) equipped with a FlatQuad 5060F silicon drift detector (SDD) as an X-ray energy dispersive spectroscopy (EDS) detector from Bruker Nano (Billerica, MA, USA) with 1.2 steradians of solid angle, making it possible to achieve high X-ray count rates even at low primary energy. A TESCAN model VEGA3 SBU EasyProbe (Brno, The Czech Republic) with a Bruker EDS detector (Billerica, MA, USA) was used at 20 kV to determine the bulk chemical composition of the S-CNHs.

## 3. Results and Discussion

**Figure 1:**
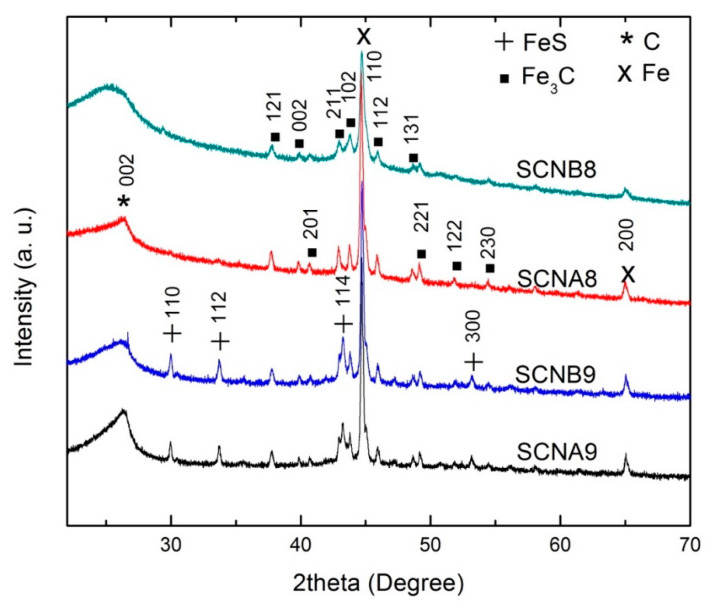
X-ray diffraction patterns of S added carbon nanohorns (S-CNHs).

Figure 1 compares the XRD patterns of the synthesized S-CNH samples. In all samples, the diffraction patterns showed evidence of graphitic structure (002) at 2θ = 26.3° with an interplanar distance d = 0.338 nm [35,36,37]. Also, the peaks corresponding to Fe_3_C were identified with planes (121), (002), (201), (211), (102), (112), (131), (221), (122), and (230). All samples showed a high-intensity peak at 2θ = 44.7° and a second characteristic peak at 2θ = 65.05° (marked by X), which corresponds to metallic Fe that remained encapsulated in the core of the carbon nanostructures due to their growth mechanisms [38]. Finally, samples SCNA9 and SCNB9 display peaks for FeS at 2θ = 29.9°, 33.7°, 43.1°, 53.1°, corresponding to the crystalline planes (110), (112), (114), and (300), respectively. The presence of FeS in these samples is attributed to the high temperature (900 °C), which favors its formation [39].

**Figure 2:**
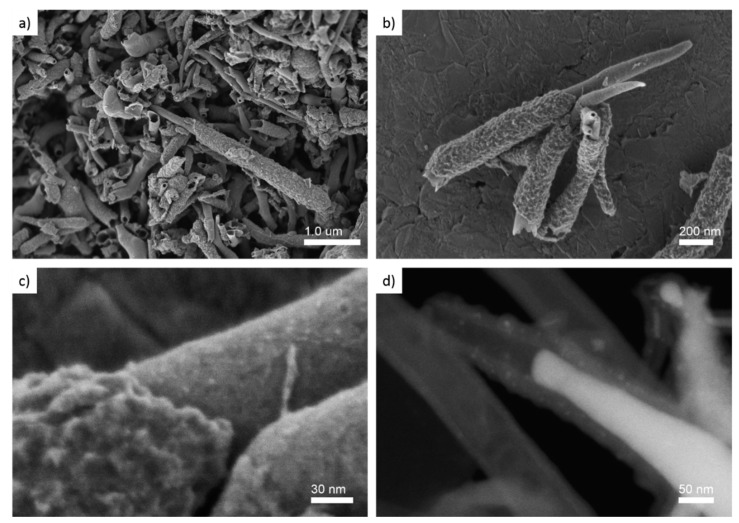
High resolution scanning electron microscopy micrographs of the SCNA9 (**a**), SCNA8 (**b**–**d**) samples. Using in-lens secondary electron detector with EL = E0 − Edecc = 2.0 − 1.5 = 0.5 kV (**a**–**c**) and dark-field transmitted electron detector (STEM-DF) at E0 = 30 kV (**d**).

The elemental composition obtained by EDS of bulk nanohorn samples shows that sulfur content was around 0.3 wt% in SCNA8 and SCNB8, and close to 1.0 wt% for SCNA9 and SCNB9 (Table S1 in the Supplementary Materials). An increase of the sulfur content is observed as the temperature of synthesis increased which is in agreement with the XRD results due to the FeS formation. The four samples presented the same morphology as revealed from a high-resolution field emission scanning electron microscope (FE-SEM) analysis. Examples of this morphology are given in Figure 2 (more HRSEM representative images of each sample are shown in the Supplementary Materials). By using a landing voltage as low as EL = E0 − Edecc = 2.0 − 1.5 = 0.5 kV (where EL is the landing voltage and Edecc is the deceleration voltage) and the high-resolution secondary electron (SE) signal collected by in-lens detectors, the surface morphology of the S-CNH was revealed. From Figure 2a,b, the horn-like structures are observed (also see Figures S1e, S2e, S3c and S4f in the Supplementary Materials). The structures have a length in the range of 1 to 3 µm and diameters in the range of from 50 to 200 nm. Along with the structures, the diameter decreases, hence showing the formation of a horn-like nanostructure. Micrographs obtained at low voltages revealed the surface textural properties of the carbon structures which present two types of layers. The outer layer exhibits a rough surface, indicating the possible presence of defects probably due to the intercalation of the sulfur atoms into the graphite lattice. On the other hand, the inside layer presents a smooth surface, a well-known characteristic of carbon nanotubes. Figure 2c is an enlarged view of the interface between the inner layer and the outer layer. In addition, Figures S1a–d, S3a–c and S4e in the Supplementary Materials show the two contrasting layers. It can be noticed that round particles of nanometer-scale (<5 nm) are unevenly dispersed at the nanohorn (Figures S1d and S3b in the Supplementary Materials) as well as at the outer layer surface (Figures S1b, S2f, S3d and S4d in the Supplementary Materials). The large scale or bulk structural patterns of the carbon nanohorn after the mechanical removal from the Vycor tube are homogenous as is observed in Figure 2a and the low magnification SEM images of the Supplementary Materials (Figures S2a,b, S3a and S4a,b).

Hollow tubular structures were observed from the dark-field scanning transmission electron microscopy (STEM-DF) obtained with E0 = 30 kV (Figure 2d, Figures S1f and S3f in the Supplementary Materials). Rod-shaped metallic nanoparticles with high atomic number contrast were also observed inside the tubular structure in Figure 2d which might be iron-based seeds from the growth process. The nanoparticles observed in Figure 2c were also noticed in the STEM-DF micrograph (Figure 2d) with the same size but their distribution looked more uniform in the outer layer.

**Figure 3:**
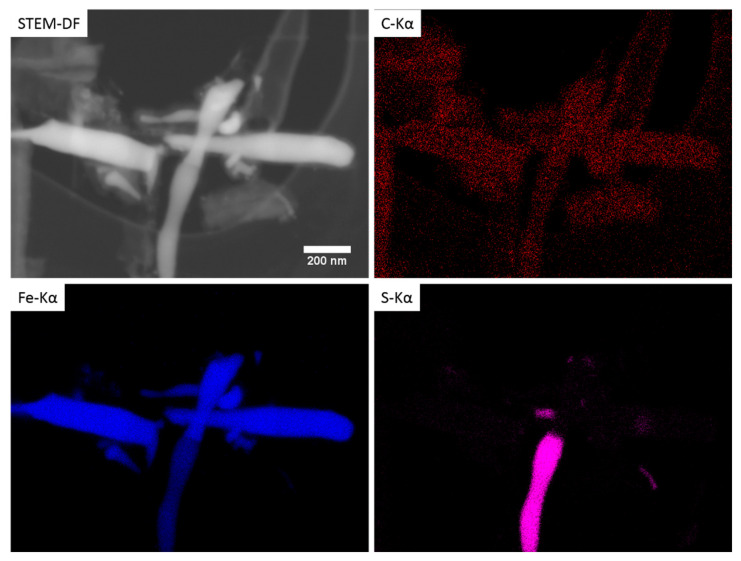
Elemental X-ray map of SCNA8 sample in STEM mode at E0 = 20 kV.

**Figure 4:**
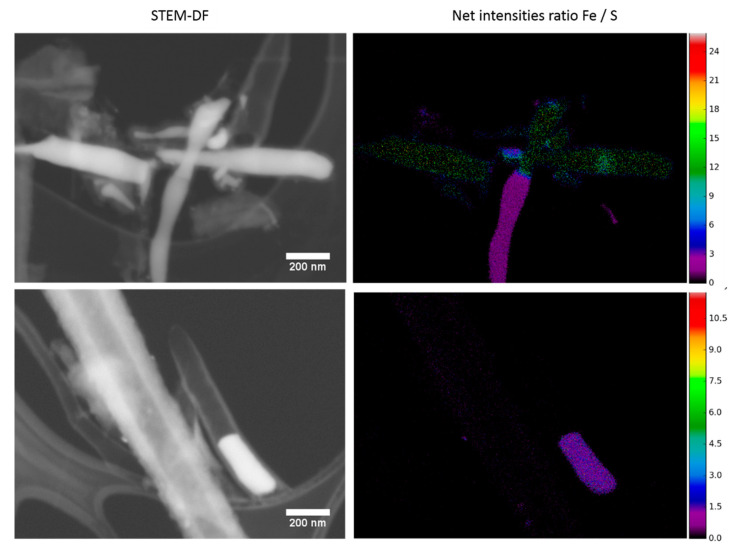
STEM-DF images (**left** side) and net intensities Fe/S ratio maps (**right** side) of the SCNA8 (**top**) and SCNA9 (**bottom**) samples at E0 = 20 kV.

An X-ray map acquired with an annular SDD detector is presented in Figure 3. It was obtained in STEM-DF mode with E0 = 20 kV on an area with both hollow and filled nanohorns. As expected, the rod-like particles inside the tubular structure were composed of a high atomic number material. From the Fe, C, and S maps, it can be deduced that these structures are either Fe_3_C or FeS which is in agreement with the XRD results. Sulfur can also be observed in the carbon area. This was confirmed by generating the net intensities Fe/S ratio maps (Figure 4), from the data used in Figure 3 in addition to that from the SCNA9 sample (Figure S5 in the ESM shows the complete X-ray map for this sample). This demonstrated that the metal particles contained inside the nanohorns were a mixture of iron carbide and iron sulfide for all samples. The samples SCNA8 and SCNB8 presented, however, a smaller amount of iron sulfide compared to iron as deduced from Figure 1. In addition, the small nanoparticles observed in Figure 2d were also observed in the outer layer of the nanohorn analyzed in Figure 4 (bottom) and were identified as a mixture of Fe and S, possibly FeS. Note that, as a result of the thick Mylar window protecting the SDD crystal from beam damage due to the backscattered electrons (BSE), the carbon signal is increased when the BSE signal increases, typically when the Fe and S signals are strong. The carbon map may thus not be fully representative of the real carbon signal.

**Figure 5:**
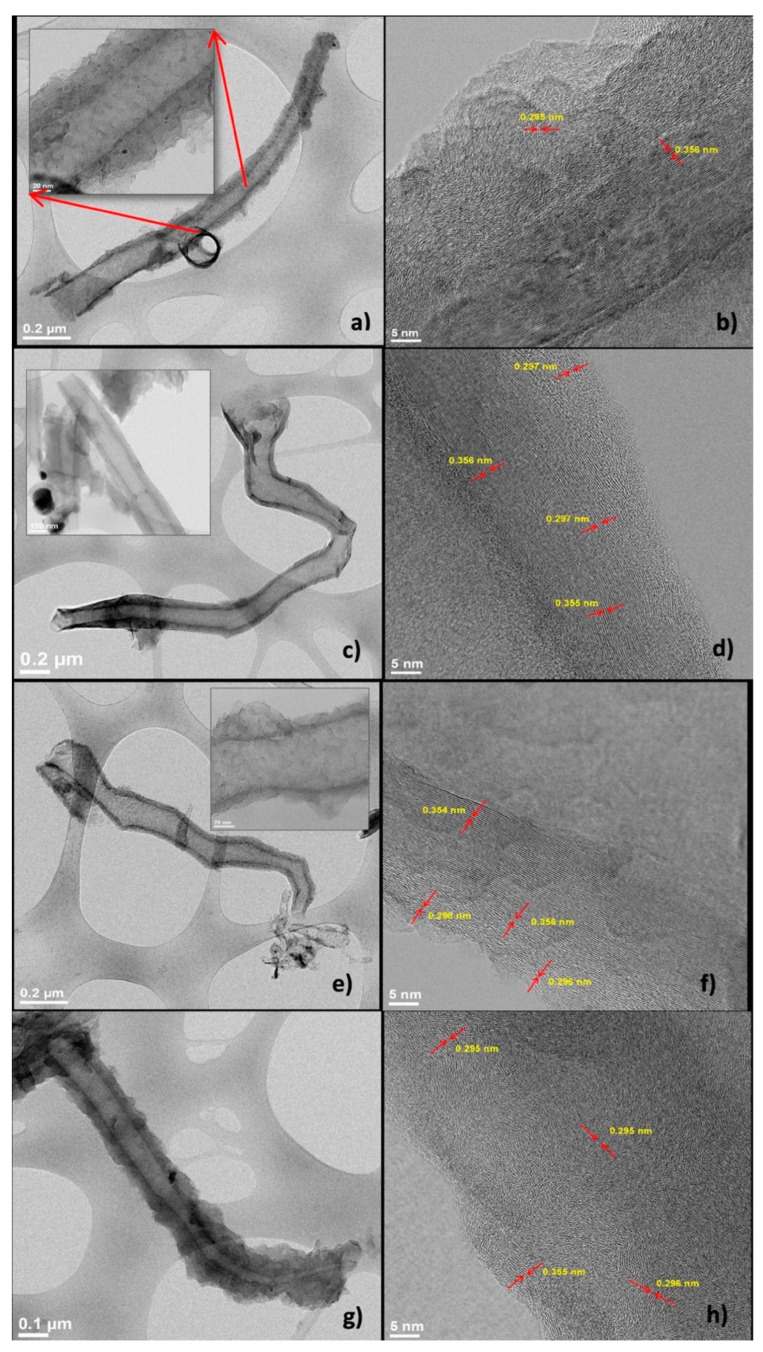
TEM images of SCNA8 (**a**,**b**), SCNA9 (**c**,**d**), SCNB8 (**e**,**f**), and SCNB9 (**g**,**h**).

Transmission electron microscopy images of SCNA8, SCNA8, SCNB8, and SCNB9 are shown in Figure 5. The microscopy images (Figure 5a,c,e,g) clearly show the presence of long tubular carbon nanostructures with diameters between 50 and 200 nm. It can be noted that, along with the structures the diameters tending to decrease, forming horn-type shapes, this behavior was observed for all the experimental conditions.

In addition, layers formed on the S-CNH external walls (ordered and amorphous) are also noticed. This behavior could be attributed to the insertion of sulfur in the structure. In the images obtained at higher magnification (Figure 5b,d,f,h), the atomic layers (lattice fringes) are evident. The lattice distances were measured in different areas of the samples. However, it was not possible to identify any clear tendency; the ordered layers (close to the core of the S-CNH), as well as the disordered layers (on top of the ordered ones), showed a mixture of lattice distances mostly of two groups, 0.355 ± 0.001 nm, and 0.296 ± 0.002 nm. Different authors have reported that the interplanar distance for graphite is in the range of 0.335 nm to 0.340 nm [40,41]. According to the XRD analysis, the first group of lattice distances is associated with modified graphite planes, possibly due to the incorporation of S. While the second group is attributed to the formation of an iron sulfide phase [42], which is in agreement with the FeS nanoparticles observed by microscopy.

**Figure 6:**
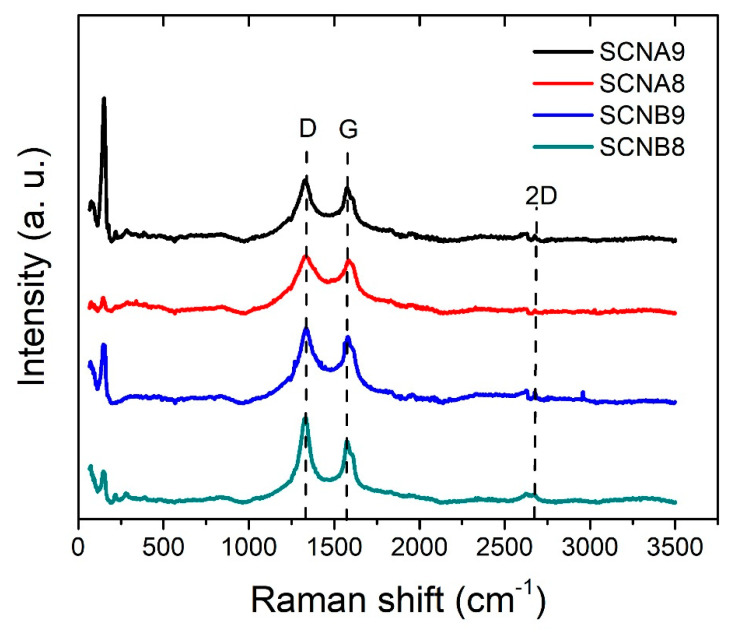
Raman spectra of sulfur-added carbon nanostructures synthesized at different temperatures and carrier gas flow rates.

The Raman spectra presented in Figure 6, show intensities that are associated with the D band (1300 cm^−1^) and G band (1600 cm^−1^). The width of the bands, present in all samples, is significantly greater than the width that is found in polycrystalline graphite [43]. Hence, the increment in the width of the peak can be attributed to the curvature of the multilayers. Moreover, the 2D band is extremely weak; a low intensity of this band was observed in MWCNT [44]. The D band is indicative of defects in the carbon layers and the intensity ratio (I_D_/I_G_) between the D and G bands provides information about the number of defects. The calculated ratio I_D_/I_G_ is 1.37 ˃ 1.16 ˃ 1.15 ˃ 1.05 for samples SCNB8, SCNA9, SCNA8, and SCNB9, respectively. A large number of defects in all of the samples may be due to the presence of sulfur in the carbon atomic lattice; also, the sulfur incorporation in the carbon network leads to a structural modification that can induce an additional band near the G band. However, there may be a slight influence of the synthesis temperature and flow rate of carrier gas on the graphitization degree. Lower synthesis temperature and low gas flow rate promote the formation of lattice defects. Finally, the Raman spectra show a group of very intense bands between 100 and 200 cm^−1^, they are mainly attributed to the radial breathing mode (RBM) associated with the inner tube’s small diameter when a suitable resonance condition is established [45]. Hence, the Raman results obtained in this band range could be attributed to the narrower part of the horns where there are a few carbon layers and small diameters, observed in the HRTEM images.

## 4. Conclusions

In summary, multiwalled carbon nanohorns with sulfur were synthesized by a single-step reaction using a modified CVD technique. The hollow carbon nanostructures have diameters that tend to decrease (in the range from 50 to 200 nm), causing horn-type shapes. During synthesis, sulfur is intercalated into the carbon lattice. Also, the S reacts with Fe forming iron sulfide nanoparticles that serve as seeds for the carbon nanostructures’ growth. High-resolution microscopy analyses show that FeS nanoparticles are detached and intercalated in the carbon lattices. The inner layers of the carbon are very well-ordered graphite layers; however, the outer layers of nanohorn surfaces have defects due to the influence of sulfur. The physical and chemical properties of this novel material are expected to be attractive for biological applications, drug carriers, adsorbents, catalysis, sensors, and energy application in electrochemical devices such as fuel cells, supercapacitors, and batteries.

## Data Availability

The data are available from the corresponding author upon reasonable request.

## Supplementary Materials

The following supporting information can be downloaded at: https://www.mdpi.com/article/10.3390/ma15103412/s1, Figure S1: High resolution scanning electron microscopy micrographs of the SCNA8 sample using the upper (secondary electrons, a, c, and e) and top (energy-filtered backscattered electrons, b and d) in-lens detectors at E0 = 0.5 kV (a–d) and E0 = 0.2 kV (e), and a dark-field transmitted electron detector (STEM-DF) at E0 = 30 kV (f); Figure S2: High-resolution scanning electron microscopy micrographs of the SCNB8 sample using the upper (secondary electrons, a, c, and e) and top (energy-filtered backscattered electrons, b, d, and f) in-lens detectors at E0 = 0.5 kV; Figure S3: High-resolution scanning electron microscopy micrographs of the SCNA9 sample using the upper (secondary electrons, a, c, and e) and top (energy-filtered backscattered electrons, b, and d) in-lens detectors at E0 = 0.5 kV, and a dark-field transmitted electron detector (STEM-DF) at E0 = 30 kV (f); Figure S4: High-resolution scanning electron microscopy micrographs of the SCNB9 sample using the upper (secondary electrons, a, c, e, and f) and top (energy-filtered backscattered electrons, b and d) in-lens detectors at E0 = 0.5 kV.; Figure S5: Elemental X-Ray map of SCNA9 sample in STEM mode at E0 = 20 kV; Table S1: Elemental Analysis (EDS) from the Bulk of the Carbon Nanohorn.
