# Supplementary material for: Structural Study of Sulfur-Added Carbon Nanohorns
Source: Materials (Basel). 2022 May 10;15(10):3412. doi: 10.3390/ma15103412 (PMC9148090; doi:10.3390/ma15103412)
Supplement: Supplementary file 1 [file materials-15-03412-s001.zip › materials-1643482-supplementary.pdf]

materials

Structural Study of Sulfur-Added Carbon Nanohorns

Ysmael Verde-Gómez [1,*], Elizabeth Montiel-Macías [1], Ana María. Valenzuela-Muñiz [1], Ivonne Alonso-Lemus [2], Mario Miki-Yoshida [3], Karim Zaghib [4], Nicolas Brodusch [5] and Raynald Gauvin [5]

1 Tecnológico Nacional de México/I.T. de Cancún, Av. Kabah km. 3, Cancún, Q.Roo, 77500, México; elizabethmontielmacias@hotmail.com (E.M.-M.); ana.vm@cancun.tecnm.mx (A.M.V.-M.)
2 CONACyT-CINVESTAV Unidad Saltillo, Sustentabilidad de los Recursos Naturales y Energía. Av. Industria Metalúrgica, Parque Industrial Saltillo-Ramos Arizpe, Ramos Arizpe, Coah., 25900, México; ivonne.alonso@cinvestav.edu.mx
3 Centro de Investigación en Materiales Avanzados S.C., Av. Miguel de Cervantes 120, Chihuahua, Chih., 31136, México; mario.miki@cimav.edu.mx
4 Department of Chemical and Materials Engineering, Concordia University, 1515 Rue Sainte-Catherine O, Montréal, QC H3G 2W1, Canada; karim.zaghib@concordia.ca
5 Department of Mining and Materials Engineering, McGill University, 3610 University Street, Montréal, QC H3A 0C5, Canada; nicolas.brodusch@mcgill.ca (N.B.); raynald.gauvin@mcgill.ca (R.G.)
* Correspondence: jose.vg@cancun.tecnm.mx; Tel.: +52-998-880-7432

materials

S1. EDS Results

Table S1. Elemental Analysis (EDS) from the Bulk of the Carbon Nanohorns.

SAMPLE	wt. %				
	C	Fe	S	O	N
SCNA8	80.0	8.7	0.3	7.5	3.3
SCNB8	77.9	6.5	0.3	12.7	2.7
SCNA9	84.9	5.1	1.1	6.0	3.4
SCNB9	81.6	11.0	1.0	6.2	0

S2. High-Resolution Scanning Electron Microscopy and Microanalysis

Figures S1 to S4 show the HRSEM micrographs of each sample using different detectors. The horn-like structures, the two layers, and iron nanoparticles (white spots in the backscattering images) can be appreciated in all samples.

materials

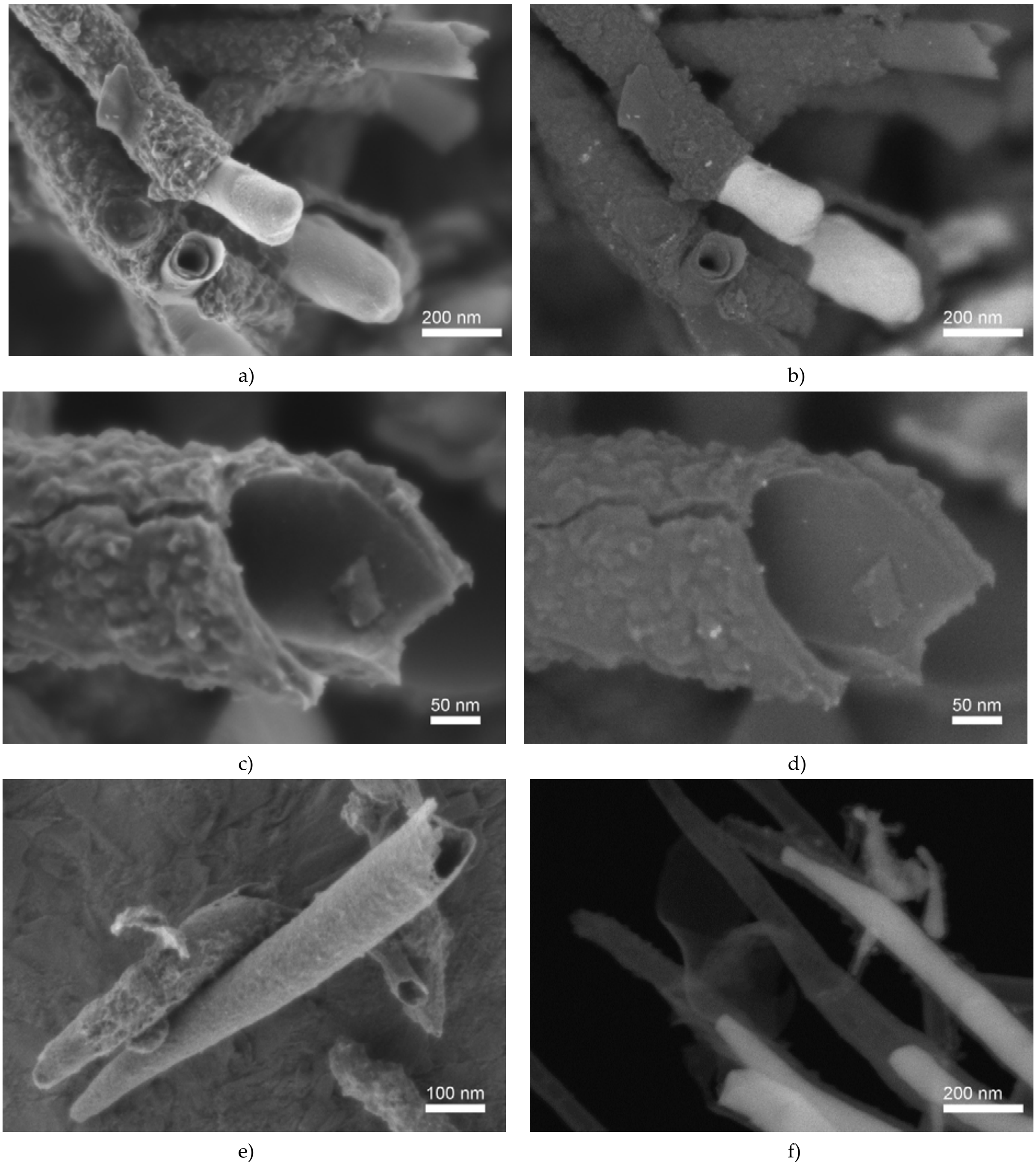

Figure S1. High-resolution scanning electron microscopy micrographs of the SCNA8 sample using the upper secondary electrons detector (Figures a, c and e) and top energy-filtered backscattered electrons (Figures b and d) in-lens detectors at E0 = 0.5 kV (a-d) and E0 = 0.2 kV (e); and a dark-field transmitted electron detector (STEM-DF) at E0 = 30 kV (f).

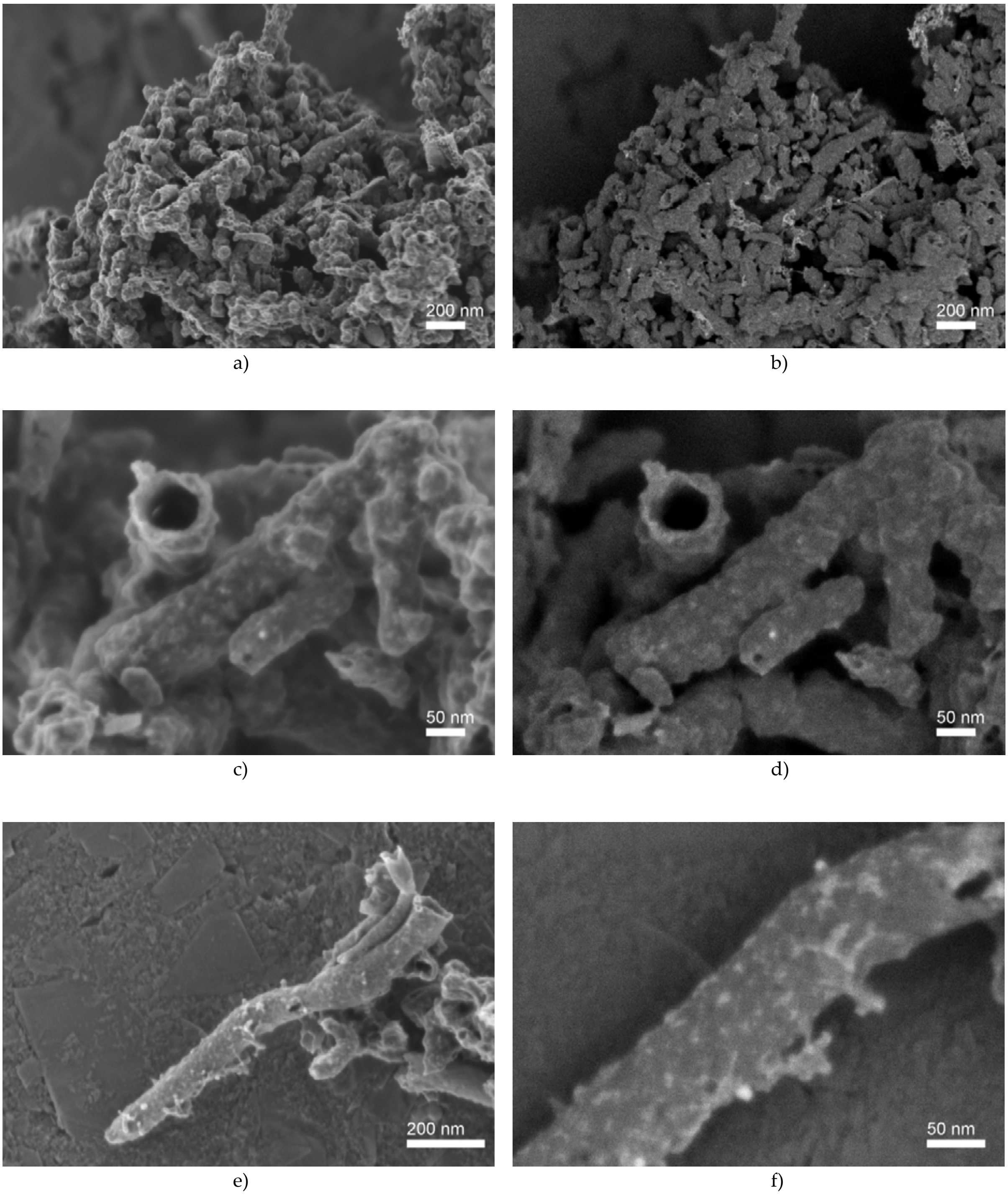

Figure S2. High-resolution scanning electron microscopy micrographs of the SCNB8 sample using the upper (secondary electrons, a, c, and e) and top (energy-filtered backscattered electrons, b, d, and f) in-lens detectors at E0 = 0.5 kV.

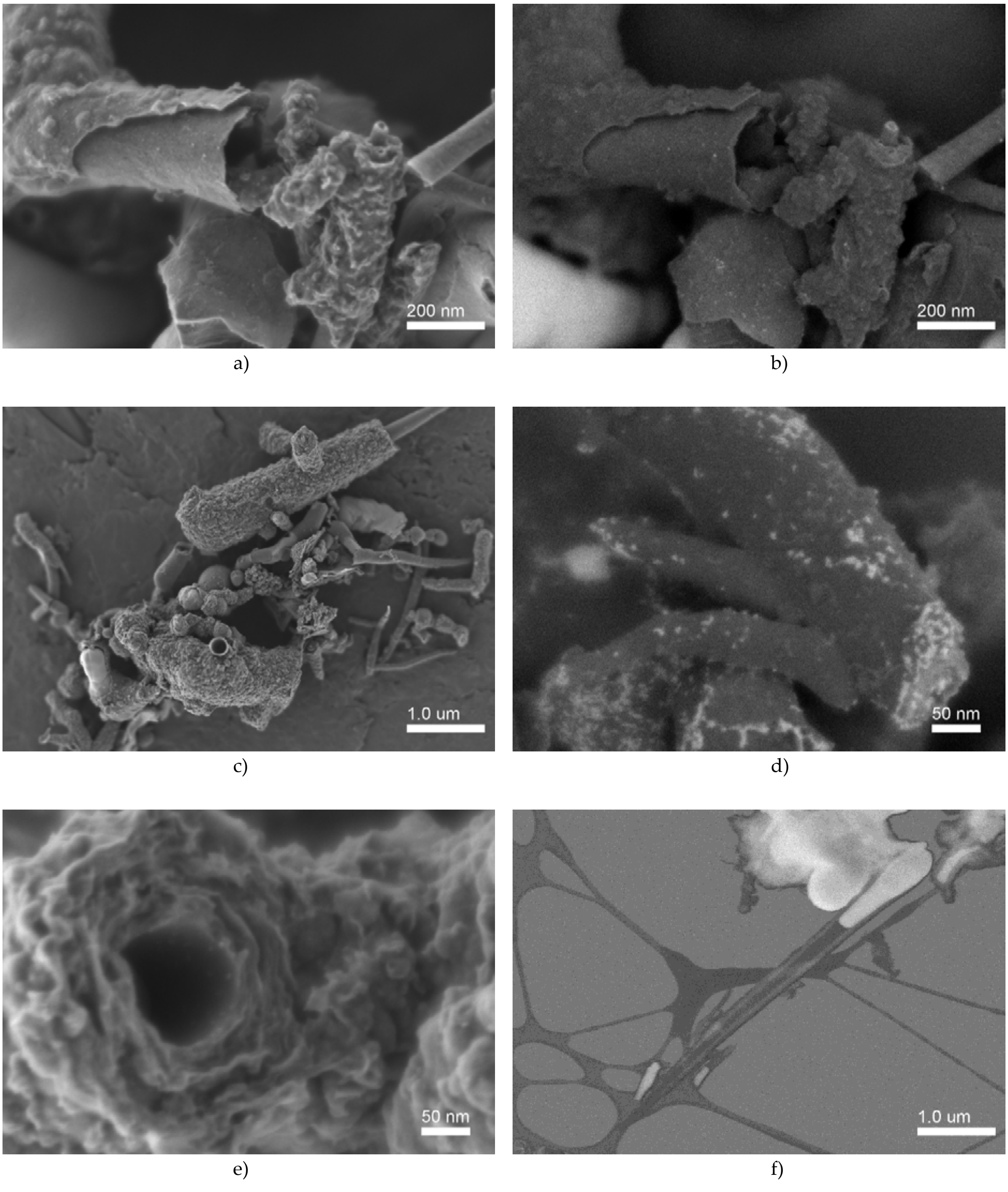

Figure S3. High-resolution scanning electron microscopy micrographs of the SCNA9 sample using the upper (secondary electrons, a, c, and e) and top (energy-filtered backscattered electrons, b, and d) in-lens detectors at E0 = 0.5 kV, and a dark-field transmitted electron detector (STEM-DF) at E0 = 30 kV (f).

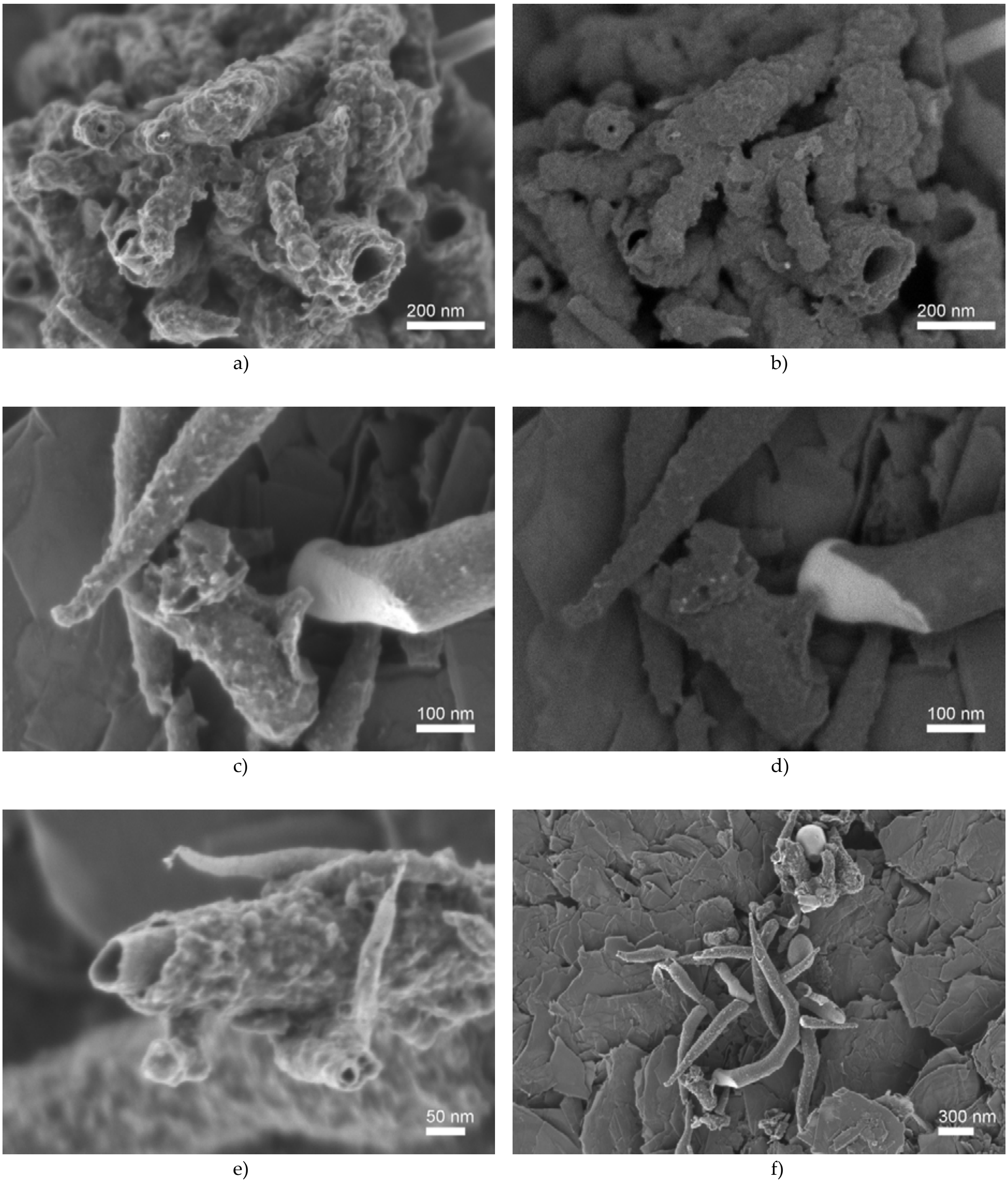

Figure S4. High-resolution scanning electron microscopy micrographs of the SCNB9 sample. using the upper (secondary electrons, a, c, e, and f) and top (energy-filtered backscattered electrons, b and d) in-lens detectors at E0 = 0.5 kV.

materials

In Figure S5, X-ray microanalysis mapping using FlatQuad EDS detector exhibit the presence of iron, carbon, and sulfur in the core of the nanohorns, suggesting the formation of iron sulfide, and iron carbide; consistent with XRD patterns.

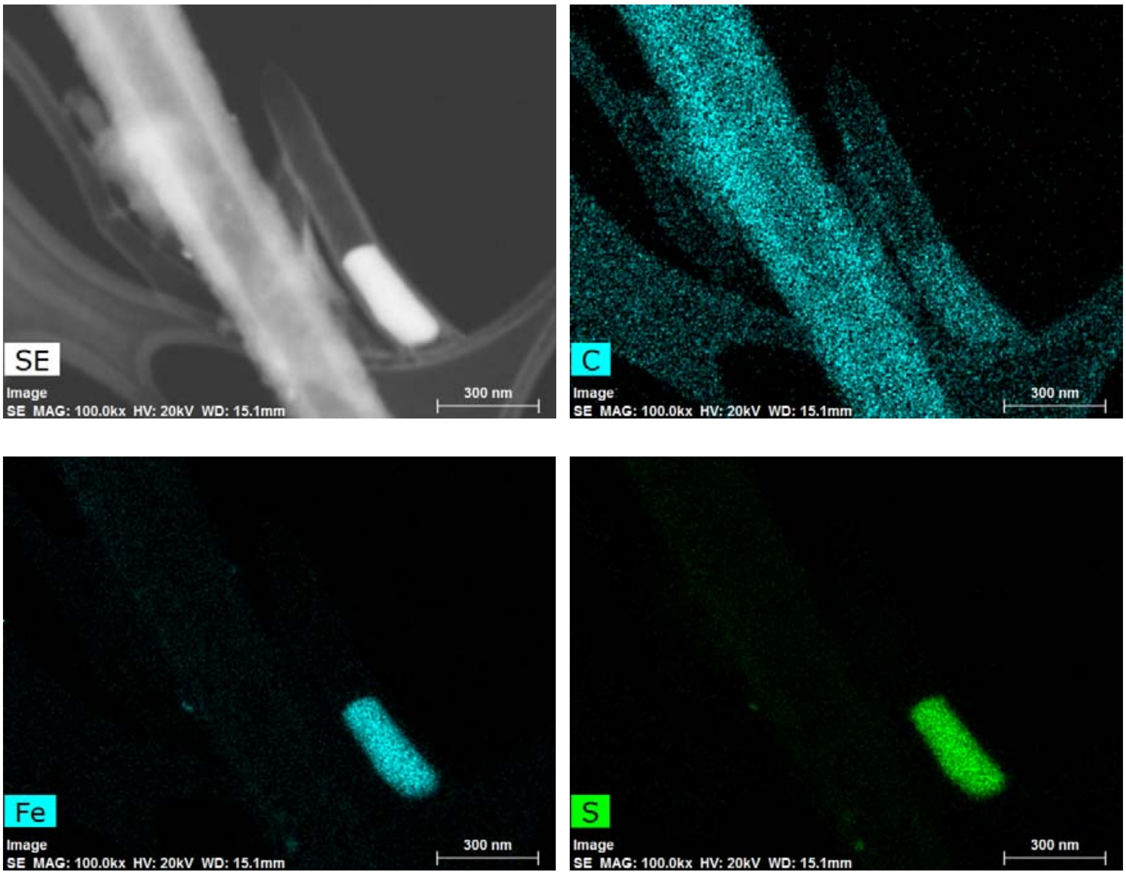

Figure S5. Elemental X-Ray map of SCNA9 sample in STEM mode at E_0 = 20 kV.
